# Multi-omics Analysis of Periodontal Pocket Microbial Communities Pre- and Posttreatment

**DOI:** 10.1128/mSystems.00016-17

**Published:** 2017-06-20

**Authors:** Katy J. Califf, Karen Schwarzberg-Lipson, Neha Garg, Sean M. Gibbons, J. Gregory Caporaso, Jørgen Slots, Chloe Cohen, Pieter C. Dorrestein, Scott T. Kelley

**Affiliations:** a Center for Microbial Genetics and Genomics and Department of Biological Sciences, Northern Arizona University, Flagstaff, Arizona, USA; b Collaborative Mass Spectrometry Innovation Center, Skaggs School of Pharmacy and Pharmaceutical Sciences, University of California, San Diego, California, USA; c Department of Biological Engineering, Massachusetts Institute of Technology, Cambridge, Massachusetts, USA; d Herman Ostrow School of Dentistry, University of Southern California, Los Angeles, California, USA; e Department of Pediatrics, University of California, San Diego, San Diego, California, USA; f Department of Biology, San Diego State University, San Diego, California, USA; University of Trento

**Keywords:** 16S rRNA, diagnostics, metabolome, microbiome, molecular networking, periodontal disease, periodontitis, shotgun metagenomics

## Abstract

Periodontitis is a polymicrobial infectious disease that causes breakdown of the periodontal ligament and alveolar bone. We employed a meta-omics approach that included microbial 16S rRNA amplicon sequencing, shotgun metagenomics, and tandem mass spectrometry to analyze sub- and supragingival biofilms in adults with chronic periodontitis pre- and posttreatment with 0.25% sodium hypochlorite. Microbial samples were collected with periodontal curettes from 3- to 12-mm-deep periodontal pockets at the baseline and at 2 weeks and 3 months. All data types showed high interpersonal variability, and there was a significant correlation between phylogenetic diversity and pocket depth at the baseline and a strong correlation (rho = 0.21; *P* = 0.008) between metabolite diversity and maximum pocket depth (MPD). Analysis of subgingival baseline samples (16S rRNA and shotgun metagenomics) found positive correlations between abundances of particular bacterial genera and MPD, including *Porphyromonas*, *Treponema*, *Tannerella*, and *Desulfovibrio* species and unknown taxon SHD-231. At 2 weeks posttreatment, we observed an almost complete turnover in the bacterial genera (16S rRNA) and species (shotgun metagenomics) correlated with MPD. Among the metabolites detected, the medians of the 20 most abundant metabolites were significantly correlated with MPD pre- and posttreatment. Finally, tests of periodontal biofilm community instability found markedly higher taxonomic instability in patients who did not improve posttreatment than in patients who did improve (UniFrac distances; *t* = −3.59; *P* = 0.002). Interestingly, the opposite pattern occurred in the metabolic profiles (Bray-Curtis; *t* = 2.42; *P* = 0.02). Our results suggested that multi-omics approaches, and metabolomics analysis in particular, could enhance treatment prediction and reveal patients most likely to improve posttreatment.

**IMPORTANCE** Periodontal disease affects the majority of adults worldwide and has been linked to numerous systemic diseases. Despite decades of research, the reasons for the substantial differences among periodontitis patients in disease incidence, progressivity, and response to treatment remain poorly understood. While deep sequencing of oral bacterial communities has greatly expanded our comprehension of the microbial diversity of periodontal disease and identified associations with healthy and disease states, predicting treatment outcomes remains elusive. Our results suggest that combining multiple omics approaches enhances the ability to differentiate among disease states and determine differential effects of treatment, particularly with the addition of metabolomic information. Furthermore, multi-omics analysis of biofilm community instability indicated that these approaches provide new tools for investigating the ecological dynamics underlying the progressive periodontal disease process.

## Abstract (precis)

Periodontal disease affects the majority of adults worldwide and has been linked to numerous systemic diseases. Despite decades of research, the reasons for the substantial differences among periodontitis patients in disease incidence, progressivity, and response to treatment remain poorly understood. While deep sequencing of oral bacterial communities has greatly expanded our comprehension of the microbial diversity of periodontal disease and identified associations with healthy and disease states, predicting treatment outcomes remains elusive. Our results suggest that combining multiple omics approaches enhances the ability to differentiate among disease states and determine differential effects of treatment, particularly with the addition of metabolomic information. Furthermore, multi-omics analysis of biofilm community instability indicated that these approaches provide new tools for investigating the ecological dynamics underlying the progressive periodontal disease process.

## INTRODUCTION

Periodontal disease represents a variety of clinical manifestations of infectious disorders affecting the tooth-supporting tissues (1). Traditionally, periodontal disease is divided into gingivitis and periodontitis (2). Gingivitis refers to inflammatory disease limited to gingiva with no loss of tooth-supporting structures and with periodontal pocket depths typically ranging from 2 to 4 mm. Periodontitis is a polymicrobial infection and an extension of gingivitis. The disease appears as a breakdown of the periodontal ligament and alveolar bone that can lead to loss of teeth (2, 3). Periodontitis affects the majority of adults worldwide (4) and may contribute to various systemic diseases, including atherosclerosis, cardiovascular disease (4, 5), diabetes (6), and rheumatoid arthritis (7), among others (8). Despite decades of research, the substantial differences among periodontitis patients in disease incidence, progressivity, and response to treatment are poorly understood.

The prevention and resolution of periodontitis depend on the antimicrobial therapy rendered, the timeliness of disease detection and intervention, the virulence and load of the pathogenic agents, and the immune status of the host (9–11). Conventional periodontal therapy includes a stabilization phase and a maintenance phase (11). Stabilization of the disease is accomplished by periodontal biofilm disruption and removal of calculus and other biofilm-retentive factors and may involve adjunctive antimicrobial medication and/or surgery (12). Long-term goals in the maintenance stage are to have patients exercise proper plaque control and commit to professional prophylactic treatments to minimize the likelihood of a clinical relapse. While conventional periodontal therapy and follow-up maintenance can be effective in reducing inflammation and restoring damaged tissue, many patients do not improve significantly and may even continue to decline posttreatment (13, 14). The addition of antibiotic therapy may improve treatment outcomes, but some patients still do not improve over the long term (15). Hurdles to the successful treatment of periodontitis include microbial community complexity (16–18) and its pathogenicity (19), as well as various protective and destructive immune responses to specific microbes or the microbial consortium (9).

The application of culture-independent molecular methods to study periodontal disease has shed new light on the true complexity of periodontal biofilms and periodontitis. Single-marker gene studies assaying bacterial diversity using small-subunit rRNA gene sequences (16S rRNA) have revealed highly complex and distinct periodontal disease communities associated with healthy and diseased pockets even within the same mouth (15–21). Other studies have revealed a pattern of extreme interpersonal variability in microbiome composition that includes dramatic differences in response to the same treatment regimen (15, 16). Shotgun metagenomic studies have also been applied to study periodontitis. Metagenomic methods provide a less biased assessment of species diversity (though with reduced depth) (22) and can be used to investigate the gene content of microbial communities in relation to disease status. For instance, metagenomic studies have suggested that the more diseased (deeper) pockets tend to be richer in metabolic pathways, have a greater abundance of virulence factors, and are poorer in biosynthesis pathways than healthy (shallower) pockets (23, 24).

Here, we report the results of a multi-omics time series analysis of periodontitis that combined high-throughput sequencing of bacterial community 16S rRNA, shotgun metagenomics, and a metabolomic analysis using tandem mass spectrometry (MS/MS). Specifically, we used these multi-omics techniques to analyze samples in a follow-up to a study investigating the use of 0.25% sodium hypochlorite (diluted bleach) on dental biofilm formation (25). While Gonzales et al. (25) demonstrated a significant clinical improvement in periodontal disease during a longitudinal 3-month study, they did not determine whether or not treatment altered the biofilm communities. Using the multi-omics approach, we determined microbial composition, functional gene content, and metabolic profiles at the baseline and how these communities shifted after 2 weeks and 3 months both within and among individuals. Overall, our study demonstrated how combining multi-omics data sets provides deeper insight into periodontal disease and showed the promise of MS/MS in particular for enhancing our understanding of periodontal biofilm dynamics.

## RESULTS

DNA was successfully extracted from a total of 218 subgingival and 73 supragingival samples from 34 individuals, and 287 of these samples from 33 of the 34 individuals produced sufficient 16S rRNA amplicon product for next-generation sequencing (NGS; see Table S1 in the supplemental material). We also selected 24 subgingival samples for shotgun metagenome library preparation (it was cost prohibitive for us to analyze all of the samples by shotgun sequencing), 23 of which produced sufficient sequence information for analysis (Table S1). Illumina sequencing generated a combined total of 8,523,044 sequences (read length, 150 bp) for all of the 16S rRNA amplicon libraries, with an average of 29,491 per library. Sequencing of metagenomic samples produced 352,668,299 reads (read length, 150 bp) before filtering, and 164,498,456 reads were left after the removal of human sequences (53% human contamination). MS/MS extractions were successful with a total of 217 samples from 25 individuals (Table S1 and S2).

10.1128/mSystems.00016-17.4 TABLE S1  Mapping file and details of sample used in multi-omics analyses. Download TABLE S1, XLSX file, 0.1 MB. Copyright © 2017 Califf et al. 2017 Califf et al. This content is distributed under the terms of the Creative Commons Attribution 4.0 International license.

10.1128/mSystems.00016-17.5 TABLE S2  Top 15 results correlated with MPD by using Spearman’s correlations for subgingival samples in each data category. Download TABLE S2, XLS file, 0.02 MB. Copyright © 2017 Califf et al. 2017 Califf et al. This content is distributed under the terms of the Creative Commons Attribution 4.0 International license.

### Alpha diversity.

Analysis of the pretreatment alpha diversity of 16S rRNA amplicon operational taxonomic units (OTUs) found a significant relationship between the maximum pocket depth (MPD) and the estimated phylogenetic diversity (PD) of communities as measured by Faith’s PD. In other words, there was a correlation between a sample collected from the deepest periodontal pocket of an individual at a particular time point, which we term the MPD, and a higher overall PD of bacterial 16S rRNA sequences in the sample (Spearman’s rank correlation; rho = 0.27, *P* = 0.01). While the correlation with MPD was strong, there was no significant correlation between 16S rRNA PD and pocket depth (rho = 0.1, *P* = 0.32). The per-sample diversity of MS^1^ metabolite features was also assessed in relationship to MPD. Since ecological alpha diversity estimates (e.g., Faith’s PD and Chao1) assume the presence of distinct, independent species-like units that have the same ancestry, such measures appeared to be inappropriate for investigating the diversity or complexity of metabolic features from MS data. Instead, we calculated the mean and median numbers of metabolic features of the pockets in an individual at a particular time point and determined whether these were correlated with MPD. Both the mean (Spearman’s rank correlation; rho = 0.21, *P* = 0.0001) and median (rho = 0.22, *P* = 0.007) numbers of metabolites were strongly correlated with MPD. The correlations of mean and median numbers of metabolites with pocket depth were also significant, but the correlations were not as strong (mean, rho = 0.15; *P* = 0.05; median, rho = 0.21, *P* = 0.008).

### Beta diversity.

**FIG 1:**
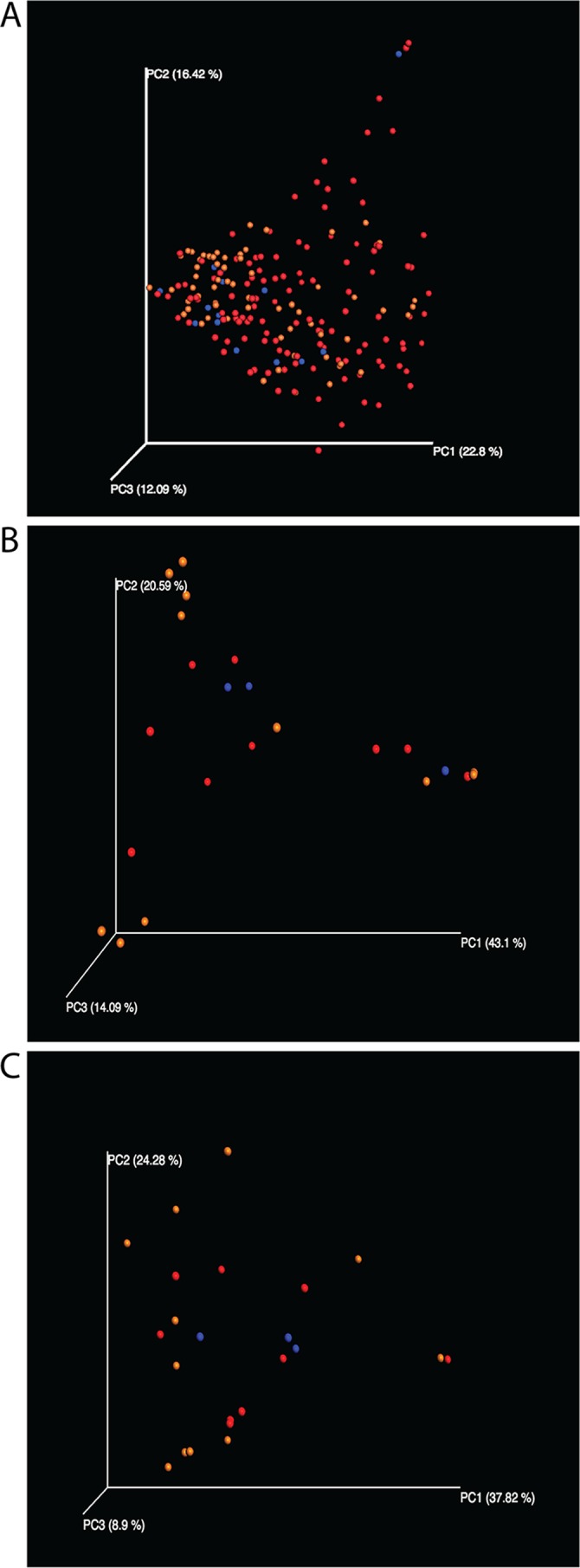
PCoA plots of subgingival samples with disease classification overlaid. Disease classifications: red, up to 6 mm; blue, >6 to 8 mm; orange, >8 mm. (A) 16S rRNA OTU-based weighted UniFrac distances (analysis of similarity, *R* = 0.015; *P* = 0.27); (B) Shotgun metagenomic species abundance-based Bray-Curtis distances (*R* = 0.006; *P* = 0.43); (C) Shotgun metagenomic pathway abundance-based Bray-Curtis distances (*R* = 0.204; *P* = 0.051).

To analyze beta diversity, intersample distances were calculated between all pairs of samples by using each of the multi-omics data sets. Weighted UniFrac and Bray-Curtis distances were calculated for the 16S rRNA sequence data, while Bray-Curtis distances were calculated for all other data sets, i.e., metagenomic shotgun species, metagenomic pathway analysis, and metabolite features. Principal-coordinate analysis (PCoA) of the pairwise distance matrices based on the subgingival (Fig. 1) or supragingival (data not shown) samples showed no clear separation of communities on the basis of disease class in any multi-omics data set. There was also no correlation between greater UniFrac distances (16S rRNA) and the absolute difference between the MPDs of samples (Mantel test; *r* = 0.01105; *P* = 0.356).

### Mantel tests.

Mantel tests were used to detect correlations between all of the various distance matrices for the subgingival samples. There was a highly significant correlation between pathway abundance and species distance matrices (*r* = 0.299, *P* = 0.002; Table 1) from the metagenomic data. 16S rRNA genera versus metagenomic species and 16S rRNA versus metabolites had the second and third highest correlation values (*r* = 0.117, *P* = 0.067; *r* = 0.103, *P* = 0.039), respectively.

**TABLE 1:** Mantel test results for Pearson’s correlations between pairwise distance matrices for subgingival samples only^a^

Matrix	16S rRNA genera (subgingival)	Shotgun species	Pathway abundance	Metabolites (subgingival)
16S rRNA genera (subgingival)	—^b^	0.117, 0.067, 0.145	0.095, 0.473, 0.628	0.103, 0.039, 0.101
Shotgun species	—	—	0.299, 0.002, 0.009	0.016, 0.838, 0.838
Pathway abundance	17	17	—	−0.048, 0.677, 0.8
Metabolites (subgingival)	124	17	17	—

a The values above the blank diagonal are Mantel *r* statistics, *P* values, and *q* values (FDR-adjusted *P* values), in that order. The values below the diagonal are the numbers of entries.

b —, not applicable.

### Supervised classification.

Supervised classification with the R package “randomForest” was performed for all of the multi-omics data types (26) to determine whether any of the data sets were able to classify the data by either disease class or MPD. The ratio of estimated generalization error to baseline error of the classifier for the subgingival samples is presented for each data set in Table 2. Random forests classified samples by disease class marginally better than random guessing with all multi-omics data sets but no better than random guessing based on MPD except when the classification was based on MS^1^ features (Table 2). Classification based on the MS/MS data was the most consistent among the data types in terms of classification, with the highest ratio when classifying by MPD (1.7) and the second highest ratio when classifying by disease class (1.6; Table 2). Supervised classification based on random forests with the same two categories was also performed for the supragingival samples, but the ratio of generalization error to baseline error did not exceed 1 (no better than random guessing) with any of the data sets (data not shown).

**TABLE 2:** Results of supervised classification of subgingival samples by using random forests^a^

16S rRNA genus or ratio	Metabolites or ratio^b^	Shotgun species or ratio
Disease class		
* Mycoplasma*	228.231–228.234_555–569	*Capnocytophaga granulosa*
Order ML615J-28^c^	697.907–697.909_220–226	TM7
* Desulfovibrio*	872.632–872.634_222–227	*Porphyromonas gingivalis*
* Filifactor*	453.356–453.359_701–706	TM7
* Porphyromonas*	705.697–705.703_220–226	*Porphyromonas gingivalis*
* Eubacterium*	689.111–689.113_218–222	*Capnocytophaga granulosa*
Family *Leptotrichiaceae*^c^	284.294–284.296_672–675	*Streptococcus cristatus*
* Desulfomicrobium*	257.246–257.249_225–227	TM7
Family *Tissierellaceae*^c^	185.113–185.115_217–222	*Leptotrichia buccalis*
* SHD*-*231*	698.306–698.308_221–227	Order *Neisseriales*^*c*^
1.5^b^	1.61404^b^	1.71429^b^
MPD		
* Desulfovibrio*	480.546–480.554_525–581	*Shuttleworthia satelles*
Family *Leptotrichiaceae*^c^	285.279–285.280_134–136	*Tannerella forsythia*
Order: ML615J-28^c^	272.294–272.297_370–376	*Riemerella^c^*
Family *Tissierellaceae*^c^	477.355–477.366_104–116	*Selenomonas artemidis*
* Butyrivibrio*	497.357–497.366_765–803	*Treponema denticola*
* Pedobacter*	382.156–382.159_330–362	*Shuttleworthia^c^*
* Megasphaera*	611.354–611.358_462–473	*Leptotrichia wadei*
* Desulfomicrobium*	285.277–285.280_149–177	*Rothia^c^*
* Porphyromonas*	497.083–497.091_349–367	*Actinomyces oris*
* Paludibacter*	475.096–475.105_713–757	Family *Aerococcaceae*^c^
1.25581^b^	1.70175^b^	0.89674^b^

a Shown are the top 10 features, as indicated by importance scores, predicting disease class (top) and MPD (bottom) from supervised classification analyses of subgingival samples.

b Ratio of estimated generalization error to baseline error of the classifier for subgingival samples.

c Not classifiable at a lower taxonomic level.

### Feature correlations.

Spearman rank correlation with false discovery rate (FDR) correction for multiple comparisons was used to determine which, if any, of the most abundant taxa (16S rRNA, metagenomics), functional pathways (metagenomics), or metabolic features (MS^1^) correlated with MPD in subgingival and supragingival samples. To minimize the total number of comparisons evaluated, we performed correlations of only the 20 most abundant taxa, pathways, or features in each data set. Table 3 summarized the results; for the full set of results for all of the data sets, see Table S4. The majority of the 16S rRNA OTU clusters were identifiable to the genus level, with a few exceptions that were only classified at the family or order level. Most of the metagenomic taxa were classifiable to the species level with MetaPhlAn2, but several were only classifiable to the family or order level. In contrast, we were unable to classify any of the metabolic features with the network pattern matching when using the Global Natural Products Social (GNPS) reference database. Thus, for the metabolomic features, we have included the precursor mass and retention time for each feature (see Table S4).

**TABLE 3:** Summary of Spearman rank correlations for subgingival features correlated with MPD pre- and posttreatment^*a*^

Data set	No. of features correlated		
	Positively	Negatively	Significantly (*P* < 0.05)^a^
Pretreatment			
16S rRNA genera	14	6	12
Metagenomes (taxa)	18	2	0
Metagenomes (pathways)	9	11	0
Metabolites	4	16	20
Posttreatment			
16S rRNA genera	12	8	11
Metagenomes (taxa)	6	14	0
Metagenomes (pathways)	7	13	0
Metabolites	17	4	20

a The top 20 results for each feature are shown. Full results are shown in Table S4.

Analysis of 16S rRNA amplicon sequences and shotgun metagenomic reads detected a significant shift in the distribution of most abundant taxa pre- and posttreatment in the subgingival samples (Table 3). At the baseline, the 16S rRNA data analysis found significant positive correlations with genera containing known periodontal pathogens, such as *Porphyromonas*, *Treponema*, *Desulfovibrio*, and *Mycoplasma* (Table S4). The shotgun metagenomic analysis identified positive correlations with *Porphyromonas* and *Tannerella* species, but also a number of *Treponema* species and other members of the class *Spirochaetes*, though the correlations were not significant after multiple-comparison correction. A number of other, more unusual genera, such as *Acholeplasma*, *Mogibacterium*, *Desulfobulbus*, and *Scardovia*, and bacteria known only through sequence analysis (no cultured representatives), such as SHD-231, TG5, and ML615J-28, were also significantly positively correlated with MPD (Table 3). Members of the bacterial genera *Eikenella*, *Methylobacterium*, and *Scardovia* were also abundant but negatively correlated with MPD.

After 2 weeks, both 16S rRNA data and shotgun metagenomics revealed dramatic shifts in the most abundant taxa (Table 3). Analysis of 16S rRNA sequences found that only the genus *Desulfovibrio* remained among the 20 most abundant taxa. In addition to a number of uncultivated genera, *Butyrivibrio*, *Methanobrevibacter*, *Pedobacter*, *Peptococcus*, and *Filifactor* were significantly positively correlated with MPD, while *Streptococcus*, *Aerococcus*, and *Slackia* became more abundant and were negatively correlated with MPD (Table S4). The posttreatment metagenomic data set was dominated by unclassified *Selenomonas* spp. and *Prevotella* and *Dialister* spp., which were all negatively correlated with MPD, while *Mogibacterium* and *Eggerthia* spp. were positively correlated with MPD.

Analysis of functional pathways in the metagenomic shotgun data and MS^1^ metabolite features also uncovered complete turnover in both pathways and metabolites abundant before and after treatment (Table 3). While none of the pathway correlations were significantly correlated with MPD either positively or negatively, all 20 of the more common feature types were correlated with MPD both before and after treatment. Also, while 16 of the 20 pretreatment features were negatively correlated with MPD, 18 of the 20 posttreatment features were positively correlated with pocket depth.

### MS^2^ analysis.

**FIG 2:**
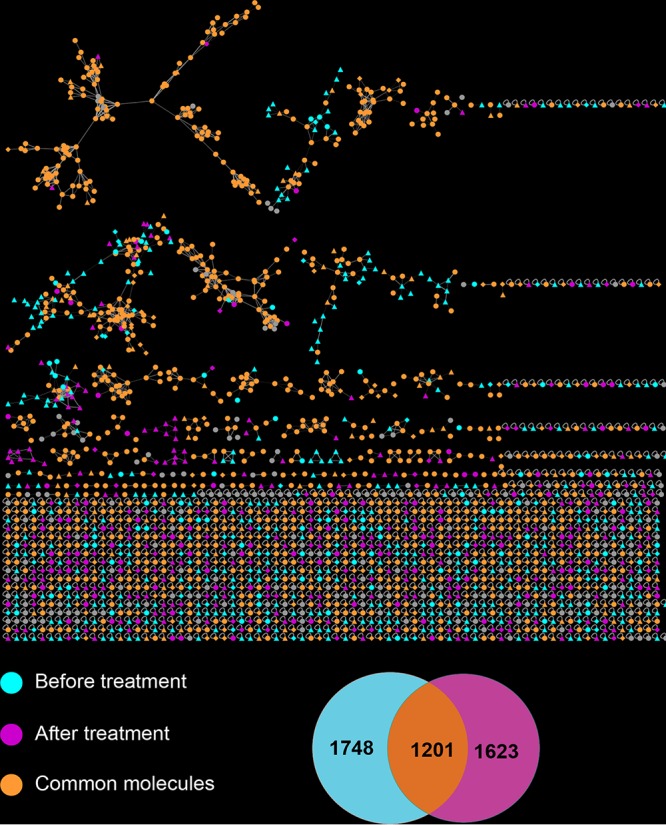
Molecular network analysis of UPLC-Q-TOF MS^2^ data. The networks were based on subgingival samples collected from the same periodontal pockets of 12 individuals before and after treatment. Individual MS^2^ features are highlighted on the basis of whether they were detected in samples collected before treatment, after treatment, or both. The Venn diagram indicates the numbers of features unique to before-treatment (blue) or after-treatment (purple) samples or common to both sets (orange).

Figures 2 and S1 illustrate the MS^2^ network results in relation to treatment and pocket depth, respectively. The MS^2^ of a molecule can be considered a type of molecular “fingerprint” that can be used to search an existing MS^2^ database of known molecules (the GNPS database) to find a match, much like the FBI matches human fingerprints. Approximately 42.9% of the total of 2,153 features determined by MS^2^ were found only in samples before or after treatment, while 57.1% of the rest were shared.

### CIS analysis.

**FIG 3:**
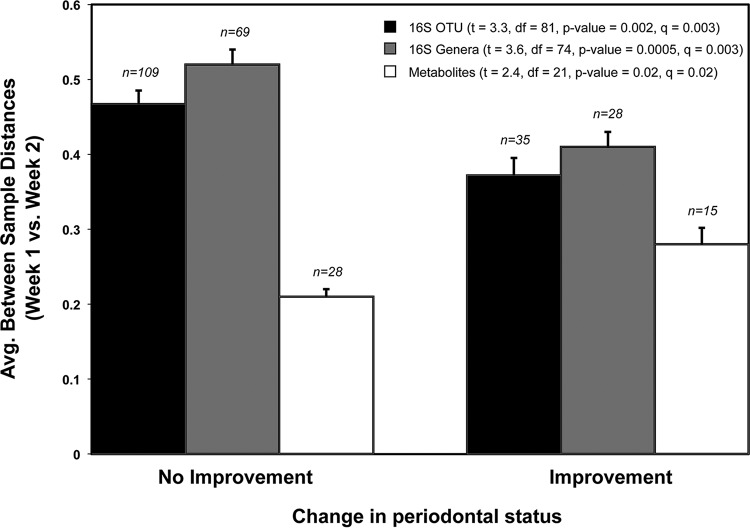
CIS analysis. Bar graph illustrating changes in the microbiome composition of individuals who improved in measurable pocket depth (≥*x* mm) between time points with treatment and individuals who did not improve or got worse with treatment. The bars indicate the average UniFrac and Bray-Curtis distances between samples of the same periodontal pocket pretreatment and 2 weeks after treatment. Sample numbers are presented above the bars. *t* tests corrected for multiple comparisons were used to compare distances between categories for each data set: 16S rRNA OTU-based distances (weighted UniFrac), 16S rRNA genus-based distances (Bray-Curtis), and MS^1^ metabolite feature-based distances (Bray-Curtis).

In the final analysis, which we termed a community instability (CIS) analysis, we use the weighted UniFrac or Bray-Curtis distances calculated between all pairs of samples to determine how “stable” particular periodontal microbial communities were over time. Under our definition of stability, greater UniFrac or Bray-Curtis distances between two samples of the same periodontal pocket at different time points indicated greater instability of the microbial community in the pocket. Using this measure, we compared the average community stability of periodontal pockets that improved after treatment with those that did not improve. A periodontal pocket was considered “improved” if the pocket depth measurement became smaller after treatment (shallower) and “not improved” if it stayed the same or increased (deeper pocket). Our analysis of instability based on the 16S rRNA data found significantly greater CIS in periodontal pockets that did not improve than in pockets that improved, while analysis of the Bray-Curtis distances calculated on the basis of the MS/MS metabolite features found the exact opposite (Fig. 3).

## DISCUSSION

The etiology of periodontal disease has proven to be especially difficult to disentangle because of both the complexity of the periodontal biofilm community and the high level of individual variability among patients in both bacterial composition and treatment outcomes. Our multi-omics analysis of patients with severe periodontal disease provided deep and nuanced insights into the complex dynamics of this polymicrobial infection. The combination of 16S rRNA marker gene sequencing and shotgun metagenomics complemented each other in the analysis of taxonomic diversity. The different data sets concurred on a number of key taxa previously implicated in periodontal disease but also highlighted other microbes that were “missed” in some data sets. Most surprisingly, however, was the strength of the patterns in the untargeted metabolomic data set. Of the three different data sets, MS diversity and abundance had the strongest associations with disease and pocket depth. Moreover, our time series analysis of the metabolomic features showed that diversity moved in the opposite direction with respect to taxonomic diversity, suggesting that the addition of MS information could add an important new dimension to our understanding of periodontal disease dynamics.

Similar to previous NGS studies of periodontal disease (16, 21), analysis of the 16S rRNA data set found a positive relationship between the within-sample PD (Faith’s PD) of subgingival samples and periodontal pocket depth (MPD) pretreatment (Spearman’s rank correlation; rho = 0.27, *P* = 0.01). In other words, the evolutionary biodiversity of the biofilm bacterial communities was generally higher in deeper periodontal pockets, which coincides with the progressive nature of the disease. The mean per-sample abundance of metabolic features (MS^1^ data) was also strongly correlated with MPD with a much lower *P* value than with the 16S rRNA data (rho = 0.21, *P* = 0.0001), suggesting that metabolite abundance may be a more sensitive indicator of community status in regard to changes in periodontal disease status.

Beta diversity analyses (between-sample comparisons of biodiversity), on the other hand, found no clear relationship between disease class and subgingival or supragingival diversity within the 16S rRNA, shotgun metagenomic, or metabolomic data sets either pre- or posttreatment (Fig. 1; data not shown). These findings run somewhat counter to those of previous studies using 16S rRNA data, which found that “healthy” sites could be distinguished from “diseased” sites on the bases of beta diversity comparisons. One explanation for this discrepancy could be the difference in classification between our study and previous studies. Many of the patients in our study had extremely deep periodontal pockets, and the healthiest category in our study included patients with periodontal pockets that would have been classified as diseased in those other studies. What was a consistent theme across the studies, however, was the high level of individual variability among patients with the same disease classification or pocket depth in all of the data sets. While having proven useful in other deep-sequencing studies to classify environments, supervised learning methods were not effective at classifying samples on the basis of disease class or MPD (Table 2).

A detailed look at the abundances of specific bacterial taxa and MS^1^ features found that numerous specific lineages correlated with MPD and also that the taxa and features correlated with MPD changed almost completely posttreatment. On the basis of the analysis of the 16S rRNA data, a number of genera containing known oral pathogens were positively correlated with MPD pretreatment (i.e., the abundances tended to be higher as MPD increased), including *Porphyromonas*, *Treponema*, and *Mycoplasma*, while other genera previously associated with a healthier periodontium were negatively correlated, including *Haemophilus* and *Eikenella* (Table 3). These results were supported by the shotgun metagenomic data, which found similar positive correlations with multiple *Porphyromonas* and *Treponema* species/strains, as well as a number of *Tannerella* species and several unknown members of the class *Spirochaetes* (Table 3). Also notable in the analysis of the 16S rRNA data was the high number of relatively rare, poorly studied, or completely unknown bacterial taxa (e.g., SHD-231, TG5, and ML615J-28) that correlated with MPD. Genera such as *Desulfovibrio*, *Acholeplasma*, *Mogibacterium*, and *Scardovia* have all been identified in the oral cavities of humans or animals and can be found in the HOMID database (27), but their specific roles in periodontal disease are unknown.

Genus- and species-level analyses of posttreatment samples showed an almost complete turnover in the 20 most abundant taxa for both the 16S rRNA and shotgun metagenomic data. In the 16S rRNA data, only *Desulfovibrio* and the unknown taxon ML615J-28 remained among the 20 most abundant genera, and many other genera, including *Streptococcus*, a genus strongly associated with greater health, became more abundant. This same pattern held with the shotgun metagenomic data, where the 20 more abundant species and strains were completely different, with many more negatively correlated with MPD posttreatment (Table 3). The low *P* values after multiple-comparison corrections for the taxonomic and pathway correlations of the shotgun metagenomic data (Table 3) are likely a result of the relatively small number of samples studied (i.e., 19).

The most surprising result of the correlation analysis was the number of significant correlations found in the 20 most abundant MS^1^ features both pre- and posttreatment. All of the top features were significantly correlated with MPD both pre- and posttreatment, and none of the features were the same in the two categories (Table 3). Furthermore, the majority of the most abundant pretreatment MS^1^ features (16 of 20) were negatively correlated with MPD, but after treatment, the majority (18 of 20) were positively correlated with MPD. These data suggest that the metabolite abundance information may provide a particularly sensitive indicator of periodontal pocket status and biofilm dynamics over time or after disturbance. Furthermore, the switch from negatively correlated to positively correlated associations in the MS^1^ data was particularly striking and may indicate a fundamental shift in the whole community’s metabolic function.

While analysis of the MS^1^ feature abundances allowed for a high degree of discriminatory power, analysis of the MS^2^ data was unable to identify these discriminatory features. Most studies that have generated MS^2^ data for complex microbial communities have also reported a very low number of matches (28, 29). For example, a recent study using multi-omics, including MS/MS data, to study the human microbiome in relation to fermented food consumption reported a 1.3% match rate of the MS/MS results (29). A 1.8% match rate is the mean rate of matching for all metabolomic data sets in the GNPS database (28). Of these, a majority were food related (e.g., gingerol from ginger root) or indicative of animal cells (e.g., cholesterol and other lipids) and none were clearly bacterial in origin.

Therefore, it is not surprising that we determined the identities of so few metabolites in our complex microbiological samples. Nevertheless, we did find some surprising patterns in the number of features that were unique to samples before treatment as opposed to after treatment (Fig. 2). Around 42.9% of the total of 2,153 features determined by MS^2^ were found only in samples before or after treatment, with 57.1% of the rest shared. Interestingly, the same pattern was not true of pocket depth (Fig. S1). In a previous study of human skin that combined MS/MS and 16S rRNA amplicon sequencing, Bouslimani et al. (30) found no correlation between bacterial diversity and metabolic features, indicating that many or most of the features detected were not bacterial in origin. In the present study, on the other hand, Mantel tests assessing the correspondence between distance matrices determined a correlation coefficient (*r*) of 0.103 (*P* = 0.039; corrected *P* = 0.101; Table 1). This correlation was nearly as strong as the one between the 16S rRNA genera and shotgun metagenomic taxon distance matrices (Table 1). However, since MPD is also correlated with bacterial alpha diversity, this make it difficult to conclude that the metabolites were of bacterial origin, especially since host-derived metabolites might also be expected to increase in deep infected pockets.

10.1128/mSystems.00016-17.3 FIG S1  Molecular network analysis of UPLC-Q-TOF MS^2^ data. The networks were based on subgingival samples collected from the same periodontal pockets of 12 individuals before and after treatment. Individual MS^2^ features are highlighted on the basis of whether they were in samples collected at specific pocket depths. Download FIG S1, PDF file, 0.8 MB. Copyright © 2017 Califf et al. 2017 Califf et al. This content is distributed under the terms of the Creative Commons Attribution 4.0 International license.

The final hypothesis we tested was whether the dynamism of the microbial communities was indicative of treatment outcome, perhaps more so than shifts in species composition. To test this hypothesis, we performed a CIS analysis that used the UniFrac, Bray-Curtis, or other community distance metrics to determine how much a community changes over time. For example, a large 16S rRNA UniFrac distance between two samples of the same periodontal pocket community indicates a greater change in the overall community composition (more “instability”) than a small UniFrac distance. Using this test, we found a striking difference in the stability of periodontal pockets that showed improvement versus those did not change or worsened: average CIS values based on the 16S rRNA data were higher for periodontal pocket communities that did not improve than for those that did improve (Fig. 3). Interestingly, a CIS analysis of the metabolomic data showed the exact opposite trend; i.e., average CIS (16S rRNA) values were lower for pockets that did not improve than for those that did improve (Fig. 3).

The correspondence between high levels of taxonomic instability and disease suggests an important role for the immune system in determining the outcome of treatment. A review by Darveau (31) posited that periodontal disease is a general disruption of immune system homeostasis by key pathogens such as *Porphyromonas gingivalis*. Thus, the higher instability of individuals who did not improve could be a reflection of a more easily disrupted homeostasis. Since periodontal disease is a complex polymicrobial infection with many pathogens and individual immune responses to specific pathogens can vary significantly, this may help explain why it has been so difficult to correlate the presence of specific pathogens or even community structures with treatment success in periodontal studies.

So, how to explain the opposite trend occurring with the metabolites? One possible explanation is that the bacterial communities of healthier pockets have a greater community function and are more responsive to disturbance over time. Ecological studies of macroscopic communities (e.g., grasslands, coral reefs, etc.) show a general relationship between diverse ecosystem function and community health and resilience. Alternatively, the greater average metabolic instability in periodontal pockets that showed improvement may be attributable to the host rather than the bacterial communities. For metabolites, the unhealthy pockets are probably inflamed and microbes are suppressed by the immune system. Host metabolites might dominate in this scenario, which would probably lead to higher stability. On the other hand, if the microbial community is active, it is probably producing a diverse array of metabolites that change constantly, resulting in lower stability.

In summary, the multi-omics approach showed that this combination of data types (marker gene analysis, metagenomics, and metabolomics) provided rich and complementary insights into the analysis of chronic periodontal disease. Particularly exciting were the high sensitivity of the MS/MS data to temporal changes in the microbial community and the fact that the shifts in MS features appeared to reflect different processes than the taxonomy-based analyses. This indicates that the MS/MS approach could be a powerful additional tool for studying periodontal disease. To better understand the progression and treatment of periodontal disease, future studies should also include a more direct analysis of the host immune system over time (e.g., blood serum and gingival cytokine analysis), include bacterial transcriptome analysis, and focus more on the origins of the metabolic features. In addition, future studies should also follow a more rigorous time series sampling protocol by sampling the same subgingival pocket from the same tooth regardless of changes in health status. Switching pockets disrupts the ability to follow changes in the biofilm over time and determine the effects of treatment by culture-independent methods. It is also important to note that using a nonconventional treatment may cause less of a disturbance of the biofilm than conventional periodontal treatment. Finally, future studies should attempt to analyze a larger number of individuals, especially by shotgun metagenomic analysis, which has become ever more feasible as the price of sequencing continues to decrease.

## MATERIALS AND METHODS

### Study participants.

The participants enrolled in this study included a total of 19 males and 15 females with a mean age of 41 years, all of whom were patients at the graduate periodontology clinic at the Ostrow School of Dentistry of the University of Southern California (USC). Patients had an average of 27 teeth. Each patient exhibited at least four separate teeth with a pocket depth of ≥6 mm. The patients were divided into a test group of 17 who received a sodium hypochlorite rinse, and a control group of 17 who received a water rinse (Tx_Ctl column, Table S1). This treatment has been described elsewhere in detail (25, 32). All study patients received a comprehensive clinical examination at the baseline (visit 1), at day 14 (visit 2), and at month 3 (visit 3). No scaling was performed before or during the study, and the patients did not use a chlorhexidine rinse. Microbiological samples from 3- to 12-mm-deep periodontal pockets and from supragingival sites were obtained of each study patient at all three study visits (Table S1). For deep periodontal pocket sampling, three teeth were sampled per patient per time point with a sterile Gracey curette per sampled tooth. Samples from individual teeth were analyzed separately. In a few patients, where the previous diseased sample sites became minimally diseased or healthy after therapy (25), other deep pockets in the same dentition were then sampled. For the supragingival sampling, one sample pooled from three teeth with the heaviest plaque accumulation was collected per patient per time point with a sterile periodontal curette. After sampling, the paper points were stored in 200 µl of 1× phosphate-buffered saline solution and frozen immediately at −80ºC at USC. Samples were shipped on dry ice to San Diego State University and stored at −80ºC until they could be processed. Clorox regular bleach (The Clorox Company, Oakland, CA) diluted with tap water served as the source of 0.25% sodium hypochlorite. Participants were asked to rinse their mouths twice weekly for 30 s with either 15 ml of a fresh solution of 0.25% sodium hypochlorite or 15 ml of water. Participants were also instructed in conventional oral hygiene, but they received no subgingival or supragingival scaling prior to the study. This study was approved by the USC Health Sciences Campus Institutional Review Board (no. HS-10-00509). All patients understood and signed informed-consent forms and Health Insurance Portability and Accountability Act documents before enrolling in this study.

### DNA extraction, PCR amplification, and NGS.

DNA extraction and amplification of 16S rRNA gene sequences in accordance with the protocols set forth by the Earth Microbiome Project (EMP) (33). Extractions were performed at a biosafety level 2 facility at San Diego State University. Samples were thawed on ice and vortexed briefly for 3 to 5 s, and 50 µl of the thawed sample was extracted with the Mo Bio PowerSoil Extraction kit (Mo Bio Laboratories, Carlsbad, CA) into a final volume of 50 µl, which was stored at −80°C until further processing. Amplicon sequencing was performed with primers designed to be massively multiplexed and cover the V4-V5 hypervariable region of the 16S rRNA gene by the standard methods outlined by the EMP (http://www.earthmicrobiome.org/emp-standard-protocols/16S/). To minimize contamination, reaction mixtures were prepared in small batches (12 to 14 samples at a time) that were discarded and tests were repeated if the reactions failed or there was evidence of contamination in the negative control. Samples were gel purified, diluted to equimolar concentrations, pooled, and then sequenced on the Illumina MiSeq platform at The Scripps Research Institute (TSRI) core sequencing facility (La Jolla, CA). Metagenomic libraries were prepared with 1 ng of genomic DNA and the Nextera XT protocol in accordance with the manufacturer’s instructions (Illumina), and the libraries were sequenced on an Illumina HiSeq platform at TSRI.

### 16S rRNA data analysis.

Sequence data were analyzed with QIIME version 1.9 by using default parameters unless otherwise noted (34). Sequences were clustered into OTUs at 97% by open-reference OTU picking with UCLUST against the Greengenes database version gg_13_8 preclustered to 97% identity (35, 36) and classified with the RDP classifier (release 11) (37) retrained on the Greengenes database preclustered at 97% identity. Representative sequences were then aligned with PyNAST (38), and a phylogenetic tree was constructed with FastTree 2 (39) for PD calculations. We also evaluated bacterial diversity within and between samples (alpha and beta diversity, respectively) with QIIME v. 1.9 by using Chao1, observed species, PD, and weighted and unweighted UniFrac (40). For the commands used, see Text S1.

10.1128/mSystems.00016-17.1 TEXT S1  QIIME analysis commands used for 16S rRNA analyses. Download TEXT S1, PDF file, 0.2 MB. Copyright © 2017 Califf et al. 2017 Califf et al. This content is distributed under the terms of the Creative Commons Attribution 4.0 International license.

### Metagenomic data analysis.

Metagenomes were obtained by shotgun sequencing of a subset of 24 samples, 23 of which produced high-quality data. These data were filtered for human contamination with KneadData (https://bitbucket.org/biobakery/kneaddata/wiki/Home). In choosing the samples for shotgun metagenomic sequencing, we attempted to sequence as many samples as possible from the same subgingival pocket of the same patient at three different time points. This was not always possible because the same teeth and pockets were not always sampled at different time points because of the protocol. There were 352,668,299 reads before filtering, and 164,498,456 reads were left after human sequences were removed (53% human contamination). The remaining bacterial reads were analyzed with the HUMAnN2 pipeline (41) to investigate microbial pathways. The metagenomes were also run through MetaPhlAn2 (42) to identify species-level taxonomic assignment in the samples. MetaPhlAn2 uses Bowtie2 (43) to align reads. HUMAnN2 aligns reads with ChocoPhlAn (a database maintained by the Segata lab at the University of Trento) with Bowtie2, as well as Diamond (44) and UniRef50 (45) for reads that were not mapped successfully with Bowtie2. The PWY codes were identified with MetaCyc (46). The three output biom files from HUMAnN2 (gene families, pathway abundance, and pathway coverage) and the output from MetaPhlAn2 were run through QIIME’s core_diversity_analyses.py for alpha and beta diversity calculations. For the commands used, see Text S2.

10.1128/mSystems.00016-17.2 TEXT S2  Metagenomic commands used for analysis of shotgun metagenomic data. Download TEXT S2, PDF file, 0.02 MB. Copyright © 2017 Califf et al. 2017 Califf et al. This content is distributed under the terms of the Creative Commons Attribution 4.0 International license.

### Metabolite extraction.

Supragingival tooth scrapings and saliva were extracted with 1 ml of 2:1:1 methanol-acetonitrile-water. The extracts containing organic solvent were vortexed briefly, sonicated for 10 min, and incubated at room temperature for 1 h. Following incubation, the extracts were centrifuged at maximum speed for 5 min. The solvent was transferred to a fresh vial and evaporated. The dried extract was stored at −80°C.

### UPLC-MS/MS analysis.

The extracted metabolites were dissolved in 80% methanol in water and analyzed with the UltiMate 3000 ultraperformance liquid chromatography (UPLC) system (Thermo Scientific) with a Kinetex 1.7-µm C_18_ reversed-phase UHPLC column (50 by 2.1 mm) and a MaXis Q-TOF (quadrupole time of flight) mass spectrometer (Bruker Daltonics) equipped with an electrospray ionization (ESI) source. The gradient employed for chromatographic separation was 2% solvent B (98% acetonitrile and 0.1% formic acid in liquid chromatography-MS grade water with solvent A as 0.1% formic acid in water) for 2 min, a ramp of 2% B to 50% B in 2.5 min, holding at 50% B for 1.5 min, a second step of 50% B to 100% B in 5 min, holding at 100% B for 1 min, 100% to 2% B in 0.5 min, and holding at 2% B for 1.5 min at a flow rate of 0.5 ml/min throughout the run. MS results were acquired in positive-ion mode in an *m*/*z* range of 50 to 2,000. An external calibration with ESI-L Low Concentration Tuning Mix (Agilent Technologies, Santa Clara, CA) was performed prior to data collection, and the internal calibrant Hexakis(1H,1H,3H-tertrafluoropropoxy)phosphazene was used throughout the runs. A capillary voltage of 4,500 V, a nebulizer gas (nitrogen) pressure of 2 × 10^5^ Pa, an ion source temperature of 200 C, a dry gas flow rate of 9 liters/min, a source temperature of 200ºC, and spectral rates of 3 Hz for MS^1^ and 10 Hz for MS^2^ were used. To acquire MS/MS fragmentation, the 10 most intense ions per MS^1^ were selected and the collision-induced dissociation (CID) energy shown in Table S3 was used. Basic stepping function was used to fragment ions at 50 and 125% of the CID calculated for each *m*/*z* from Table S3 with a timing of 50% for each step. Similarly, basic stepping of collision peak-to-peak radio frequency voltages of 550 and 800 V with a timing of 50% for each step and transfer time stepping of 57 and 90 µs was employed. The MS/MS active-exclusion parameter was set to 3 to exclude the fragmentation of precursor ions once three spectra per precursor ion were collected. This exclusion parameter was released after 30 s. The mass of the internal calibrant was excluded from the MS^2^ list.

10.1128/mSystems.00016-17.6 TABLE S3  Collision-induced energies used for tandem MS data collection. Download TABLE S3, XLS file, 0.1 MB. Copyright © 2017 Califf et al. 2017 Califf et al. This content is distributed under the terms of the Creative Commons Attribution 4.0 International license.

10.1128/mSystems.00016-17.7 TABLE S4  Spearman rank correlations for subgingival samples with MPD (top 20 results). A plus or minus sign indicates the direction of correlation. Significant *q* values (<0.05) are highlighted in green (+) and blue (−). *, different strain of the same bacterial species. Download TABLE S4, DOCX file, 0.1 MB. Copyright © 2017 Califf et al. 2017 Califf et al. This content is distributed under the terms of the Creative Commons Attribution 4.0 International license.

### MS/MS feature analyses.

Calculations of the diversity and abundance of sample metabolomic features were based on the precursor MS^1^ scan (the MS^1^ data), which records the mass/charge ratio of all of the ions detected in that scan (30). From that value, the MS^1^ abundance of each molecule was extracted (feature detection shown by parent mass and retention time), and these data were used for all of the statistical analyses in this study (e.g., correlation analyses, supervised learning, and Mantel tests). The MS^2^ results generated during data-dependent acquisition were used to create molecular networks and Venn diagrams. Molecular networks were created by using the GNPS database online workflow at http://gnps.ucsd.edu, and the data set was used to search various MS/MS libraries available in the GNPS database by using the same workflow. The data set is publically available at the online MassIVE repository of the GNPS database under MassIVE ID number MSV000078894. The molecular network color coded by pre- or posttreatment is available at http://gnps.ucsd.edu/ProteoSAFe/status.jsp?task=a38df2291f814d2fb3d2d8779f191047. The molecular networking color coded by various pocket depths is available at http://gnps.ucsd.edu/ProteoSAFe/status.jsp?task=99735d22369942d5a85832ee9e99d12d.

### Nucleotide sequence(s).

The 16S rRNA sequences determined in this study have been deposited in the European Nucleotide Archive under project PRJEB19122 (http://www.ebi.ac.uk/ena/data/view/PRJEB19122). The shotgun metagenomic sequence data are available to the public at MG-RAST under accession numbers mgm4730562.3 to mgm4730585.3 (http://metagenomics.anl.gov/mgmain.html?mgpage=project&project=mgp21311).
