# Supplementary figures and images for: Multi-omics Analysis of Periodontal Pocket Microbial Communities Pre- and Posttreatment
Source: mSystems. 2017 Jun 20;2(3):e00016-17. doi: 10.1128/mSystems.00016-17 (PMC5513737; doi:10.1128/mSystems.00016-17)

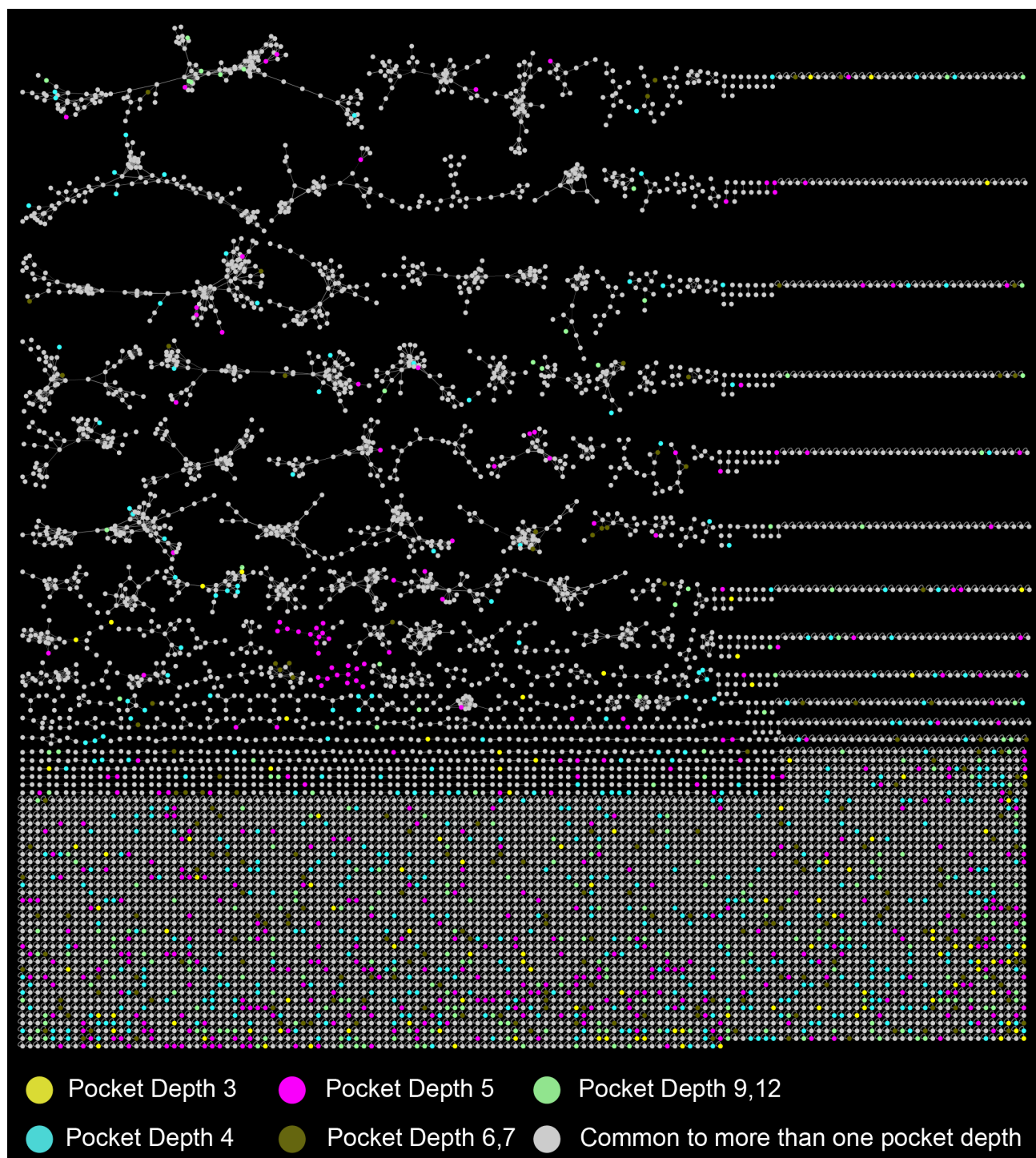

Figure S1

Supplement: FIG S1 [file sys003172112sf1.pdf]
