# Supplementary material for: Multi-omics Analysis of Periodontal Pocket Microbial Communities Pre- and Posttreatment
Source: mSystems. 2017 Jun 20;2(3):e00016-17. doi: 10.1128/mSystems.00016-17 (PMC5513737; doi:10.1128/mSystems.00016-17)
Supplement: TEXT S1 [file sys003172112s6.pdf]

QIIME Analyses

16S Analysis

2 sequencing runs:
- 5/10/14
- 6/19/14

Final mapping file to be used for ALL analyses:
clorox_perio_map_no_dupl_051115_FIXED.txt

For each folder, run split_libraries_fastq.py with R1 file (forward reads) as input (-i) and index file as -b.

Made shell script for each of these two sequencing runs with correct version of following split libraries (demultiplexing) command & run on monsoon:

1: KC_May2014.sh:

srun split_libraries_fastq.py -o /scratch-lt/kc2296/Periodontal_Data/16S_raw_data/slout/ -i /scratch-lt/kc2296/Periodontal_Data/16S_raw_data/051014_S0_L001_R1_001.fastq.gz -b /scratch-lt/kc2296/Periodontal_Data/16S_raw_data/051014_S0_L001_I1_001.fastq.gz --rev_comp_mapping_barcodes -m /scratch-lt/kc2296/Periodontal_Data/clorox_perio_map_no_dupl_051115_FIXED.txt

2: KC_June2014.sh:

srun split_libraries_fastq.py -o /scratch-lt/kc2296/Periodontal_Data/16S_raw_data/slout/ -i /scratch-lt/kc2296/Periodontal_Data/16S_raw_data/6_19_14_S0_L001_R1_001.fastq.gz -b /scratch-lt/kc2296/Periodontal_Data/16S_raw_data/6_19_14_S0_L001_I1_001.fastq.gz --rev_comp_mapping_barcodes -m /scratch-lt/kc2296/Periodontal_Data/clorox_perio_map_no_dupl_051115_FIXED.txt

Next, did open reference OTU picking in iterative mode:
```
pick_open_reference_otus.py -i /scratch-lt/kc2296/Periodontal_Data/16S_data/slout_May/seqs.fna,/scratch-lt/kc2296/Periodontal_Data/16S_data/slout_June/seqs.fna -o /scratch-lt/kc2296/Periodontal_Data/16S_data/or_otus/ -a -O 24
```

Summarize biom table & pick sampling depth:
```
biom summarize-table -i otu_table_mc2_w_tax_no_pynast_failures.biom -o 16S_summary.txt
```

Will use **6628** as sampling depth.

Next: Core diversity analysis:

First: remove 1 sample from 1/8/13:
`filter_samples_from_otu_table.py -i` /scratch-lt/kc2296/Periodontal_Data/16S_data/or_otus/otu_table_mc2_w_tax_no_pynast_failures.biom -o /scratch-lt/kc2296/Periodontal_Data/16S_data/or_otus/16S_otu_table_NoJan.biom --sample_id_fp /scratch-lt/kc2296/Periodontal_Data/16S_data/or_otus/Jan_sampleID.txt --negate_sample_id_fp

the following is in 'KJC_periodCD.sh' within /scratch-lt/kc2296/Periodontal_Data/16S_data/Core_Diversity/:

srun core_diversity_analyses.py -i /scratch-lt/kc2296/Periodontal_Data/16S_data/or_otus/16S_otu_table_NoJan.biom -o /scratch-lt/kc2296/Periodontal_Data/16S_data/Core_Diversity/Results/ -e 6628 -m /scratch-lt/kc2296/Periodontal_Data/clorox_perio_map_no_dupl_051115_FIXED.txt -a -O 24 -t /scratch-lt/kc2296/Periodontal_Data/16S_data/or_otus/rep_set.tre

zip results:
```
tar -czf Core_Diversity_16S.tgz Results/
```

Evenly sampled biom table given as output from Core Diversity Analysis that will be used for all other downstream analysis: **table_even6628.biom.gz**

→ unzipped: gunzip **table_even6628.biom**

To get 16S bray curtis distance matrix:

beta_diversity_through_plots.py -i /scratch-lt/kc2296/Periodontal_Data/16S_data/or_otus/16S_otu_table_NoJan.biom -m /scratch-lt/kc2296/Periodontal_Data/16S_data/clorox_perio_map_no_dupl_051115_FIXED.txt -e 6628 -p /scratch-lt/kc2296/Periodontal_Data/Metabolite_Data/beta_diversity_params.txt -o /scratch-lt/kc2296/Periodontal_Data/16S_data/16S_bray_curtis_beta_div_results/

To get PCoA plot for pre-treatment individuals only:

```
filter_samples_from_otu_table.py -i 16S_otu_table_NoJan.biom -o
16S_otu_table_NoJan_preTMTonly.biom -m /scratch-
lt/kc2296/Periodontal_Data/clorox_perio_map_no_dupl_051115_FIXED.txt -
s 'TimePoint:1'

beta_diversity_through_plots.py -i
16S_data/or_otus/16S_otu_table_NoJan_preTMTonly.biom -m
clorox_perio_map_no_dupl_051115_FIXED_preTMTonly.txt -e 6628 -p
```

```
beta_diversity_params.txt -t 16S_data/or_otus/rep_set.tre -o
preTMT_beta_div/
```

zip results:

```
tar -czf PreTMT_Beta_Diversity_16S.tgz preTMT_beta_div/
```

clorox_perio_map_no_dupl_051115_FIXED_preTMTonly.txt

PA testing only

-made mapping file with only 'pa' samples →
clorox_perio_map_no_dupl_051115_FIXED_pa_ONLY.txt
(*had to remove one individual for which there were missing data ("NA") for DiseasClass)

*Cannot do paired diff testing w/only pa samples, because the only variable left (Disease class) is categorical

→ Core diversity with 'pa' samples only:

```
filter_samples_from_otu_table.py -i /scratch-
lt/kc2296/Periodontal_Data/16S_data/or_otus/16S_otu_table_NoJan.biom -
o /scratch-
lt/kc2296/Periodontal_Data/16S_data/or_otus/16S_pa_only.biom -m
/scratch-
lt/kc2296/Periodontal_Data/16S_data/clorox_perio_map_no_dupl_051115_FI
XED.txt -s 'SampleSite:pa'
```

□ Use 13501 for even sampling for this biom table

```
core_diversity_analyses.py -i /scratch-
lt/kc2296/Periodontal_Data/16S_data/or_otus/16S_pa_only.biom -e 13501
-m /scratch-
lt/kc2296/Periodontal_Data/16S_data/clorox_perio_map_no_dupl_051115_FI
XED.txt -a -O 24 -t /scratch-
lt/kc2296/Periodontal_Data/16S_data/or_otus/rep_set.tre
```

zip results:

```
tar -czf Core_Diversity_pa_only.tgz pa_CoreDiv_Results/
```

Metabolite Analysis

→ Most current metabolite 'OTU table' (i.e. bucket table) received from Neha 6 Feb 2015:
Saliva_Tooth.xls

→ edited Sample IDs to match those in the mapping file as shown in Metabolite_Sample_IDs (Old Saliva Tooth file is saved on katy's computer as Saliva_Tooth_original_IDs.txt_

→ added '#OTU_ID' to cell A1

→ OTUs/Metabolites will be problematic for QIIME

i.e.: 943.990-943.994 25-239, 944.990-945.000 25-239

Replaced spaces with underscores and commas with semi-colons

→ **Removed these two following sample IDs** from the OTU table (Saliva_tooth.txt), because Neha said they did not have seq data (emailed Scott 2x for confirmation, never heard back):

- 12.2by_3_RA11_01_23904.mzXML
- 19.3bx_GC2_01_24036.mzXML

→ Duplicate row in Saliva_Tooth.xls:

- 257.248-257.249_593-596;_258.251-258.253_593-596: in cells 6232 **and** 6796: **DELETED row 6796**

New IDs & deleted samples saved to Saliva_tooth.txt

Saliva_tooth.txt was changed to a biom table and the name was altered:

biom convert -i Saliva_Tooth.txt -o Metabolite_OTU_table.biom --table-type="OTU table" --to-json

Pulled out all the samples from the earlier date (5/10/14): There is also 1 sample in this mapping file from 1/8/13; pulling that sample out as well.

-Seem to have trouble filtering by date, so made a list of June sample IDs, and filtered by that:

filter_samples_from_otu_table.py -i Metabolite_OTU_table.biom -o Metabolite_OTU_table_June_only.biom --sample_id_fp /scratch-lt/kc2296/Periodontal_Data/Metabolite_Data/June_IDs.txt

biom summarize-table -i Metabolite_OTU_table_June_only.biom -o June_summ.txt

From this summary, chose even sampling depth as: 15743156 (lowest #)

<u>Alpha Diversity analysis:</u>

alpha_diversity.ph -i /scratch-lt/kc2296/Periodontal_Data/Metabolite_Data/Metabolite_OTU_table_June_only.biom -o Metabolite_Alpha_Diversity_Results -m observed_otus,shannon,simpson

<u>Beta Diversity analysis:</u>

beta_diversity_through_plots.py -i /scratch-lt/kc2296/Periodontal_Data/Metabolite_Data/Metabolite_OTU_table_June_only.biom -o Beta_plots -e 15643156 -m /scratch-lt/kc2296/Periodontal_Data/16S_data/Metabolite_mapping_PairedDiffs_PC1noNA_noPA.txt -p beta_diversity_params.txt

beta_diversity_through_plots.py -i /scratch-lt/kc2296/Periodontal_Data/Metabolite_Data/Metabolite_OTU_table_June_only.biom -o Metabolite_Beta_Diversity_Results -e 15743156 -m /scratch-lt/kc2296/Periodontal_Data/16S_data/clorox_perio_map_no_dupl_051115_FIXED.txt -p beta_diversity_params.txt

beta_diversity_through_plots.py -i /scratch-lt/kc2296/Periodontal_Data/Metabolite_Data/Metabolite_OTU_table_June_only.biom -o Metabolite_Beta_Diversity_Results -e 15743156 -m /scratch-lt/kc2296/Periodontal_Data/16S_data/clorox_perio_map_no_dupl_051115_FIXED.txt -p beta_diversity_params.txt

<u>Compare 16S vs metabolite hellinger distance matrices:</u>

-pull out saliva data first, from both 16S and metabolite data:

16S:
filter_distance_matrix.py -i /scratch-lt/kc2296/Periodontal_Data/16S_data/16S_beta_hellinger_results/hellinger_dm.txt -m /scratch-lt/kc2296/Periodontal_Data/16S_data/clorox_perio_map_no_dupl_051115_FIXED.txt -s "SampleSite:pa" --negate -o /scratch-lt/kc2296/Periodontal_Data/16S_data/16S_hellinger_dm_noPA.txt

filter_distance_matrix.py -i /scratch-lt/kc2296/Periodontal_Data/16S_data/16S_bray_curtis_beta_div_results/bray_curtis_dm.txt -m /scratch-lt/kc2296/Periodontal_Data/16S_data/clorox_perio_map_no_dupl_051115_FIXED.txt -s "SampleSite:pa" --negate -o /scratch-lt/kc2296/Periodontal_Data/16S_data/16S_bray_curtis_dm_noPA.txt

Metabolite data:
filter_distance_matrix.py -i /scratch-lt/kc2296/Periodontal_Data/Metabolite_Data/Metabolite_Beta_Diversity_Results/bray_curtis_dm.txt -m /scratch-lt/kc2296/Periodontal_Data/16S_data/clorox_perio_map_no_dupl_051115_FIXED.txt -s "SampleSite:pa" --negate -o /scratch-lt/kc2296/Periodontal_Data/Metabolite_Data/Metabolite_bray_curtis_dm_noPA.txt

filter_distance_matrix.py -i /scratch-lt/kc2296/Periodontal_Data/Metabolite_Data/Metabolite_Beta_Diversity_Hellinger_Results/hellin

ger_dm.txt -m /scratch-lt/kc2296/Periodontal_Data/16S_data/clorox_perio_map_no_dupl_051115_FIXED.txt -s "SampleSite:pa" --negate -o /scratch-lt/kc2296/Periodontal_Data/Metabolite_Data/Metabolite_Hellinger_dm_noPA.txt

Compare metabolite vs 16S, hellinger distance matrices:

compare_distance_matrices.py --method mantel -i /scratch-lt/kc2296/Periodontal_Data/16S_data/16S_hellinger_dm_noPA.txt,/scratch-lt/kc2296/Periodontal_Data/Metabolite_Data/Metabolite_Hellinger_dm_noPA.txt -o 16s_metabolite_Hellinger_mantel_out -n 999

Compare metabolite vs 16S, bray curtis distance matrices:

compare_distance_matrices.py --method mantel -i /scratch-lt/kc2296/Periodontal_Data/16S_data/16S_bray_curtis_dm_noPA.txt,/scratch-lt/kc2296/Periodontal_Data/Metabolite_Data/Metabolite_bray_curtis_dm_noPA.txt -o 16s_metabolite_BrayCurtis_mantel_out -n 999

Results from both: 16S vs Metabolite Mantel results

Do samples from the same individual cluster together?

First, remove 'pa'(saliva) samples from 16S unifrac distance matrices:
unweighted unifrac: filter_distance_matrix.py -i /scratch-lt/kc2296/Periodontal_Data/16S_data/Core_Diversity/Results/bdiv_even6628/unweighted_unifrac_dm.txt -m /scratch-lt/kc2296/Periodontal_Data/16S_data/clorox_perio_map_no_dupl_051115_FIXED.txt -s "SampleSite:pa" --negate -o /scratch-lt/kc2296/Periodontal_Data/16S_data/Core_Diversity/Results/bdiv_even6628/unweighted_unifrac_dm_noPA.txt

weighted unifrac: filter_distance_matrix.py -i /scratch-lt/kc2296/Periodontal_Data/16S_data/Core_Diversity/Results/bdiv_even6628/weighted_unifrac_dm.txt -m /scratch-lt/kc2296/Periodontal_Data/16S_data/clorox_perio_map_no_dupl_051115_FIXED.txt -s "SampleSite:pa" --negate -o /scratch-lt/kc2296/Periodontal_Data/16S_data/Core_Diversity/Results/bdiv_even6628/weighted_unifrac_dm_noPA.txt

Do samples from the same individual cluster together?
unweighted unifrac: compare_categories.py -i /scratch-lt/kc2296/Periodontal_Data/16S_data/Core_Diversity/Results/bdiv_even6628/unweighted_unifrac_dm_noPA.txt --method mrpp -m /scratch-

lt/kc2296/Periodontal_Data/16S_data/clorox_perio_map_no_dupl_051115_FIXED.txt -c individual -o MRPP_unweighted_16S_out -n 999

weighted unifrac: compare_categories.py -i /scratch-lt/kc2296/Periodontal_Data/16S_data/Core_Diversity/Results/bdiv_even6628/weighted_unifrac_dm_noPA.txt --method mrpp -m /scratch-lt/kc2296/Periodontal_Data/16S_data/clorox_perio_map_no_dupl_051115_FIXED.txt -c individual -o MRPP_weighted_16S_out -n 999

Metabolite:

compare_categories.py -i /scratch-lt/kc2296/Periodontal_Data/Metabolite_Data/Metabolite_bray_curtis_dm_noPA.txt --method mrpp -m /scratch-lt/kc2296/Periodontal_Data/16S_data/clorox_perio_map_no_dupl_051115_FIXED.txt -c individual -o MRPP_Metabolite_out -n 999

SourceTracker Analysis

-Filter out OTUs that are in < 1% of the samples:
filter_otus_from_otu_table.py -i /scratch-lt/kc2296/Periodontal_Data/16S_data/Core_Diversity/Results/table_even6628.biom -o /scratch-lt/kc2296/Periodontal_Data/16S_data/Core_Diversity/Results/filtered_1percent_otu_table.biom -s 3

Before: 284 samples; 32139 observations After: 270 samples; 5987 observations

-Convert BIOM table to txt:
biom convert -i filtered_1percent_otu_table.biom -o filtered_1percent_otu_table.txt --table-type "OTU table" --to-tsv

-Add 2 columns to mapping file for SourceTracker: "SourceSink" and "Env"
- Teeth as 'source'; saliva as 'sink'
- ENV = Description

Added new mapping file to monsoon: clorox_perio_map_no_dupl_051115_FIXED_SourceTracker

Bacteria of interest from Karen:
paired difference testing w/genus level otu table
do w/just 7 indiv w/just these bacteria at the genus level

1. Fusobacterium

k__Bacteria;p__Fusobacteria;c__Fusobacteriia;o__Fusobacteriales;f__Fusobacteriaceae;g__Fusobacterium

identify_paired_differences.py -m clorox_perio_map_no_dupl_051115_FIXED_paired_diffs_SEVEN.txt -o paired_diffs_specific_taxa/ -t TimePoint -s 'T1T3_samePocket:T' -x 1,3 --metadata_categories "Fusobacterium" -c individual

Ten indivs:
identify_paired_differences.py -m clorox_perio_map_no_dupl_051115_FIXED_paired_diffs_taxa_TEN.txt -o paired_diffs_specific_taxa_ten_Fuso/ -t TimePoint -x 1,2 --metadata_categories "Fusobacterium" -c individual

Ten indivs, no PA:
identify_paired_differences.py -m clorox_perio_map_no_dupl_051115_FIXED_paired_diffs_taxa_TEN_noPA.txt -o paired_diffs_specific_taxa_ten_Fuso_noPA/ -t TimePoint -x 1,2 --metadata_categories "Fusobacterium" -c individual

2. Prevotella

k__Bacteria;p__Bacteroidetes;c__Bacteroidia;o__Bacteroidales;f__Prevotellaceae;g__Prevotella

identify_paired_differences.py -m clorox_perio_map_no_dupl_051115_FIXED_paired_diffs_SEVEN.txt -o paired_diffs_specific_taxa/ -t TimePoint -s 'T1T3_samePocket:T' -x 1,3 --metadata_categories "Prevotella" -c individual

Ten indivs:
identify_paired_differences.py -m clorox_perio_map_no_dupl_051115_FIXED_paired_diffs_taxa_TEN.txt -o paired_diffs_specific_taxa_ten_Prev/ -t TimePoint -x 1,2 --metadata_categories "Prevotella" -c individual

Ten indivs, no PA:
identify_paired_differences.py -m clorox_perio_map_no_dupl_051115_FIXED_paired_diffs_taxa_TEN_noPA.txt -o paired_diffs_specific_taxa_ten_Prev_noPA/ -t TimePoint -x 1,2 --metadata_categories "Prevotella" -c individual

3. Veillonella

k__Bacteria;p__Firmicutes;c__Clostridia;o__Clostridiales;f__Veillonellaceae;g__Veillonella

identify_paired_differences.py -m clorox_perio_map_no_dupl_051115_FIXED_paired_diffs_SEVEN.txt -o paired_diffs_specific_taxa/ -t TimePoint -s 'T1T3_samePocket:T' -x 1,3 --metadata_categories "Veillonella" -c individual

Ten indivs:
identify_paired_differences.py -m clorox_perio_map_no_dupl_051115_FIXED_paired_diffs_taxa_TEN.txt -o paired_diffs_specific_taxa_ten_Veill/ -t TimePoint -x 1,2 --metadata_categories "Veillonella" -c individual

Ten indivs, no PA:
identify_paired_differences.py -m
clorox_perio_map_no_dupl_051115_FIXED_paired_diffs_taxa_TEN_noPA.txt -o
paired_diffs_specific_taxa_ten_Veill_noPA/ -t TimePoint -x 1,2 --metadata_categories
"Veillonella" -c individual

4. Streptococcus

k__Bacteria;p__Firmicutes;c__Bacilli;o__Lactobacillales;f__Streptococcaceae;g__Streptococcus

identify_paired_differences.py -m clorox_perio_map_no_dupl_051115_FIXED_paired_diffs_SEVEN.txt -o
paired_diffs_specific_taxa/ -t TimePoint -s 'T1T3_samePocket:T' -x 1,3 --metadata_categories
"Streptococcus" -c individual

Ten indivs:
identify_paired_differences.py -m
clorox_perio_map_no_dupl_051115_FIXED_paired_diffs_taxa_TEN.txt -o
paired_diffs_specific_taxa_ten_Strep/ -t TimePoint -x 1,2 --metadata_categories "Streptococcus"
-c individual

Ten indivs, no PA:
identify_paired_differences.py -m
clorox_perio_map_no_dupl_051115_FIXED_paired_diffs_taxa_TEN_noPA.txt -o
paired_diffs_specific_taxa_ten_Strep_noPA/ -t TimePoint -x 1,2 --metadata_categories
"Streptococcus" -c individual

5. Treponema

k__Bacteria;p__Spirochaetes;c__Spirochaetes;o__Spirochaetales;f__Spirochaetaceae;g__Treponema

identify_paired_differences.py -m clorox_perio_map_no_dupl_051115_FIXED_paired_diffs_SEVEN.txt -o
paired_diffs_specific_taxa/ -t TimePoint -s 'T1T3_samePocket:T' -x 1,3 --metadata_categories
"Treponema" -c individual

Ten indivs:
identify_paired_differences.py -m
clorox_perio_map_no_dupl_051115_FIXED_paired_diffs_taxa_TEN.txt -o
paired_diffs_specific_taxa_ten_Trep/ -t TimePoint -x 1,2 --metadata_categories "Treponema" -c
individual

Ten indivs, no PA:
identify_paired_differences.py -m
clorox_perio_map_no_dupl_051115_FIXED_paired_diffs_taxa_TEN_noPA.txt -o
paired_diffs_specific_taxa_ten_Trep_noPA/ -t TimePoint -x 1,2 --metadata_categories
"Treponema" -c individual

6. Porphyromonas

k__Bacteria;p__Bacteroidetes;c__Bacteroidia;o__Bacteroidales;f__Porphyromonadaceae;g__Porphyromonas

identify_paired_differences.py -m clorox_perio_map_no_dupl_051115_FIXED_paired_diffs_SEVEN.txt -o paired_diffs_specific_taxa/ -t TimePoint -s 'T1T3_samePocket:T' -x 1,3 --metadata_categories "Porphyromonas" -c individual

Ten indivs:
identify_paired_differences.py -m clorox_perio_map_no_dupl_051115_FIXED_paired_diffs_taxa_TEN.txt -o paired_diffs_specific_taxa_ten_Porph/ -t TimePoint -x 1,2 --metadata_categories "Porphyromonas" -c individual

Ten indivs, no PA:
identify_paired_differences.py -m clorox_perio_map_no_dupl_051115_FIXED_paired_diffs_taxa_TEN_noPA.txt -o paired_diffs_specific_taxa_ten_Porph_noPA/ -t TimePoint -x 1,2 --metadata_categories "Porphyromonas" -c individual

7. Selenomonas

k__Bacteria;p__Firmicutes;c__Clostridia;o__Clostridiales;f__Veillonellaceae;g__Selenomonas

identify_paired_differences.py -m clorox_perio_map_no_dupl_051115_FIXED_paired_diffs_SEVEN.txt -o paired_diffs_specific_taxa/ -t TimePoint -s 'T1T3_samePocket:T' -x 1,3 --metadata_categories "Selenomonas" -c individual

Ten indivs:
identify_paired_differences.py -m clorox_perio_map_no_dupl_051115_FIXED_paired_diffs_taxa_TEN.txt -o paired_diffs_specific_taxa_ten_Selen/ -t TimePoint -x 1,2 --metadata_categories "Selenomonas" -c individual

Ten indivs, no PA:
identify_paired_differences.py -m clorox_perio_map_no_dupl_051115_FIXED_paired_diffs_taxa_TEN_noPA.txt -o paired_diffs_specific_taxa_ten_Selen_noPA/ -t TimePoint -x 1,2 --metadata_categories "Selenomonas" -c individual

8. Leptotrichia

k__Bacteria;p__Firmicutes;c__Clostridia;o__Clostridiales;f__Veillonellaceae;g__Selenomonas

identify_paired_differences.py -m clorox_perio_map_no_dupl_051115_FIXED_paired_diffs_SEVEN.txt -o paired_diffs_specific_taxa/ -t TimePoint -s 'T1T3_samePocket:T' -x 1,3 --metadata_categories "Leptotrichia" -c individual

identify_paired_differences.py -m clorox_perio_map_no_dupl_051115_FIXED_paired_diffs_taxa_TEN.txt -o paired_diffs_specific_taxa_ten_Lepto/ -t TimePoint -x 1,2 --metadata_categories "Leptotrichia" -c individual

Ten indivs, no PA:
identify_paired_differences.py -m clorox_perio_map_no_dupl_051115_FIXED_paired_diffs_taxa_TEN_noPA.txt -o paired_diffs_specific_taxa_ten_Lepto_noPA/ -t TimePoint -x 1,2 --metadata_categories "Leptotrichia" -c individual

9. Synergistes: Genus not in our data

10. Tannerella

k__Bacteria;p__Bacteroidetes;c__Bacteroidia;o__Bacteroidales;f__Porphyromonadaceae;g__Tannerella

identify_paired_differences.py -m clorox_perio_map_no_dupl_051115_FIXED_paired_diffs_SEVEN.txt -o paired_diffs_specific_taxa/ -t TimePoint -s 'T1T3_samePocket:T' -x 1,3 --metadata_categories "Tannerella" -c individual

identify_paired_differences.py -m clorox_perio_map_no_dupl_051115_FIXED_paired_diffs_taxa_TEN.txt -o paired_diffs_specific_taxa_ten_Tann/ -t TimePoint -x 1,2 --metadata_categories "Tannerella" -c individual

Ten indivs, no PA:
identify_paired_differences.py -m clorox_perio_map_no_dupl_051115_FIXED_paired_diffs_taxa_TEN_noPA.txt -o paired_diffs_specific_taxa_ten_Tann_noPA/ -t TimePoint -x 1,2 --metadata_categories "Tannerella" -c individual

Treatment individuals only:
identify_paired_differences.py -m clorox_perio_map_no_dupl_051115_FIXED_paired_diffs_taxa_FIVE_PD_no_missing.txt -o paired_diffs_specific_taxa_Tann_noPA_tmt_only/ -t TimePoint -x 1,2 --metadata_categories "Tannerella" -c individual

11. Bacteroidetes

k__Bacteria;p__Bacteroidetes;c__Bacteroidia;o__Bacteroidales;f__Bacteroidaceae;g__Bacteroides

identify_paired_differences.py -m clorox_perio_map_no_dupl_051115_FIXED_paired_diffs_SEVEN.txt -o paired_diffs_specific_taxa/ -t TimePoint -s 'T1T3_samePocket:T' -x 1,3 --metadata_categories "Bacteroidetes" -c individual

identify_paired_differences.py -m clorox_perio_map_no_dupl_051115_FIXED_paired_diffs_taxa_TEN.txt -o paired_diffs_specific_taxa_ten_Bact/ -t TimePoint -x 1,2 --metadata_categories "Bacteroidetes" -c individual

Ten indivs, no PA:
identify_paired_differences.py -m clorox_perio_map_no_dupl_051115_FIXED_paired_diffs_taxa_TEN_noPA.txt -o paired_diffs_specific_taxa_ten_Bact_noPA/ -t TimePoint -x 1,2 --metadata_categories "Bacteroidetes" -c individual

identify_paired_differences.py -m clorox_perio_map_no_dupl_051115_FIXED_paired_diffs_taxa_TEN.txt -o paired_diffs_specific_taxa_ten_Desulf/ -t TimePoint -x 1,2 --metadata_categories "Desulfovibrio" -c individual

Ten indivs, no PA:
identify_paired_differences.py -m clorox_perio_map_no_dupl_051115_FIXED_paired_diffs_taxa_TEN_noPA.txt -o paired_diffs_specific_taxa_ten_Desulf_noPA/ -t TimePoint -x 1,2 --metadata_categories "Desulfovibrio" -c individual

identify_paired_differences.py -m clorox_perio_map_no_dupl_051115_FIXED_paired_diffs_SEVEN_d_noPA.txt -o paired_diffs_specific_taxa_7_Fuso_noPA/ -t TimePoint -s 'T1T3_samePocket:T' -x 1,3 --metadata_categories "Fusobacterium" -c individual

identify_paired_differences.py -m clorox_perio_map_no_dupl_051115_FIXED_paired_diffs_SEVEN_d_noPA.txt -o paired_diffs_specific_taxa_7_Prev_noPA/ -t TimePoint -s 'T1T3_samePocket:T' -x 1,3 --metadata_categories "Prevotella" -c individual

identify_paired_differences.py -m clorox_perio_map_no_dupl_051115_FIXED_paired_diffs_SEVEN_d_noPA.txt -o paired_diffs_specific_taxa_7_Veill_noPA/ -t TimePoint -s 'T1T3_samePocket:T' -x 1,3 --metadata_categories "Veillonella" -c individual

identify_paired_differences.py -m clorox_perio_map_no_dupl_051115_FIXED_paired_diffs_SEVEN_d_noPA.txt -o paired_diffs_specific_taxa_7_strep_noPA/ -t TimePoint -s 'T1T3_samePocket:T' -x 1,3 --metadata_categories "Streptococcus" -c individual

identify_paired_differences.py -m
clorox_perio_map_no_dupl_051115_FIXED_paired_diffs_SEVEN_d_noPA.txt -o
paired_diffs_specific_taxa_trep_noPA/ -t TimePoint -s 'T1T3_samePocket:T' -x 1,3 --
metadata_categories "Treponema" -c individual

identify_paired_differences.py -m
clorox_perio_map_no_dupl_051115_FIXED_paired_diffs_SEVEN_d_noPA.txt -o
paired_diffs_specific_taxa_7_porph_noPA/ -t TimePoint -s 'T1T3_samePocket:T' -x 1,3 --
metadata_categories "Porphyromonas" -c individual

identify_paired_differences.py -m
clorox_perio_map_no_dupl_051115_FIXED_paired_diffs_SEVEN_d_noPA.txt -o
paired_diffs_specific_taxa_7_Selen_noPA/ -t TimePoint -s 'T1T3_samePocket:T' -x 1,3 --
metadata_categories "Selenomonas" -c individual

identify_paired_differences.py -m
clorox_perio_map_no_dupl_051115_FIXED_paired_diffs_SEVEN_d_noPA.txt -o
paired_diffs_specific_taxa_7_lepto_noPA/ -t TimePoint -s 'T1T3_samePocket:T' -x 1,3 --
metadata_categories "Leptotrichia" -c individual

identify_paired_differences.py -m
clorox_perio_map_no_dupl_051115_FIXED_paired_diffs_SEVEN_d_noPA.txt -o
paired_diffs_specific_taxa_7_tann_noPA/ -t TimePoint -s 'T1T3_samePocket:T' -x 1,3 --
metadata_categories "Tannerella" -c individual

identify_paired_differences.py -m
clorox_perio_map_no_dupl_051115_FIXED_paired_diffs_SEVEN_d_noPA.txt -o
paired_diffs_specific_taxa_7_bact_noPA/ -t TimePoint -s 'T1T3_samePocket:T' -x 1,3 --
metadata_categories "Bacteroidetes" -c individual

identify_paired_differences.py -m
clorox_perio_map_no_dupl_051115_FIXED_paired_diffs_SEVEN_d_noPA.txt -o
paired_diffs_specific_taxa_7_defulf_noPA/ -t TimePoint -x 1,2 --metadata_categories
"Desulfovibrio" -c individual

pre-treatment only:
-mantel: unifrac vs. pocket depth / max pd

MaxPD:
distance_matrix_from_mapping.py -i
clorox_perio_map_no_dupl_051115_FIXED_no_MaxPD_NAs.txt -o Max_PD_dm.txt -c Max_PD

filter_distance_matrix.py -i Max_PD_dm.txt -o Max_PD_dm_PreTMTonly.txt -m
clorox_perio_map_no_dupl_051115_FIXED_noNA.txt -s 'TimePoint:1'

Unweighted unifrac:

```
compare_distance_matrices.py --method mantel -i
MaxPD_core_div/bdiv_even6628/unweighted_unifrac_dm.txt,Max_PD_dm
_PreTMTonly.txt -o mantel_out_MaxPD_unw -n 9999
```

→ non-significant

Weighted unifrac:

```
compare_distance_matrices.py --method mantel -i
MaxPD_core_div/bdiv_even6628/weighted_unifrac_dm.txt,Max_PD_dm_P
reTMTonly.txt -o mantel_out_MaxPD_wtd -n 9999
```

→ non-significant

PocketDepth:

distance_matrix_from_mapping.py -i
clorox_perio_map_no_dupl_051115_FIXED_noPD_NAs.txt -o PD_dm.txt -c PocketDepth

filter_distance_matrix.py -i PD_dm.txt -o PD_dm_PreTMTonly.txt -m
clorox_perio_map_no_dupl_051115_FIXED_noPD_NAs.txt -s 'TimePoint:1'

Unweighted unifrac:

```
compare_distance_matrices.py --method mantel -i
/Users/katycaliff/Documents/Periodontal_data/16S_data/PD_dm_PreT
MTonly.txt,unweighted_unifrac_dm.txt -o mantel_out_PD_unw -n
9999
```

→ non-significant

Weighted unifrac:

```
compare_distance_matrices.py --method mantel -i
/Users/katycaliff/Documents/Periodontal_data/16S_data/PD_dm_PreT
MTonly.txt,weighted_unifrac_dm.txt -o mantel_out_PD_wtd -n 9999
```

→ non-significant
