# Supplementary material for: Multi-omics Analysis of Periodontal Pocket Microbial Communities Pre- and Posttreatment
Source: mSystems. 2017 Jun 20;2(3):e00016-17. doi: 10.1128/mSystems.00016-17 (PMC5513737; doi:10.1128/mSystems.00016-17)
Supplement: TEXT S2 [file sys003172112s7.pdf]

Metagenomic Data Analysis Commands.

List of commands:

1. Each metagenome was run through KneadData to filter out human contamination in this fashion:
   kneaddata --input sample1.fastq --reference-db Homo_sapiens_Bowtie2_v0.1 --output sample1_kneaded
2. Each filtered metagenome was then run through HUMAnN2:
   humann2 --input sample1_kneaded/sample1_kneaddata.fastq --output sample1_humann2 --metaphlan metaphlan2/1.0 --output-format biom --remove-stratified-output --threads 5
3. All 3 biom file outputs (genefamilies, pathway abundance and pathway coverage) from the samples were then merged using QIIME's merge_otu_tables.py:
   merge_otu_tables.py -i sample1_humann2/sample1_genefamilies.biom,sample2_humann2/sample2_genefamilies.biom,...,sample23_humann2/sample23_genefamilies.biom -o genefamilies.biom
4. The biom files were converted to TSV format using biom tools:
   biom convert -i genefamilies.biom -o genefamilies.tsv --to-tsv --table-type="OTU table"
5. Each TSV file had float values converted to integers using code written by Lauren McIver at the Huttenhower lab:
   table_float_to_int.py -i genefamilies.tsv -o genefamilies_int.tsv
6. Each file was then converted back to biom format:
   biom convert -i genefamilies_int.tsv -o genefamilies_int.biom --to-json --table-type="OTU table"
7. After looking at the depth needed:
   biom summarize-table -i genefamilies_int.biom -o genefamily_int_summary.txt
8. The resulting files were then run through QIIME's core_diversity_analyses.py using the appropriate mapping file and depth (e) determined by the biom file summary:
   core_diversity_analyses.py -i genefamilies_int.biom -o core_diversity_genefamilies -m mapping.txt -e 1046761 --nonphylogenetic_diversity
