# Supplementary material for: Multi-omics Analysis of Periodontal Pocket Microbial Communities Pre- and Posttreatment
Source: mSystems. 2017 Jun 20;2(3):e00016-17. doi: 10.1128/mSystems.00016-17 (PMC5513737; doi:10.1128/mSystems.00016-17)
Supplement: TABLE S4 [file sys003172112st5.docx]

**TABLE S4** Spearman rank correlations for subgingival samples with maximum pocket depth (top 20 results). Sign (+/-) indicates direction of correlation. Significant q-values (<0.05) highlighted in green (+) and blue (-). *= Different strain of same bacterial species.

| **16S Genera** |  |  |  |
| --- | --- | --- | --- |
| Pre-Treatment |  | Post-Treatment |  |
| *Porphyromonas* | + | *Desulfovibrio* | + |
| *Desulfovibrio* | + | Order: RF39 (unclassified genus) | + |
| *SHD-231* | + | Family: Cardiobacteriaceae (unclassified genus) | + |
| *Treponema* | + | Family: Leptotrichiaceae (unclassified genus) | + |
| *Haemophilus* | - | *Streptococcus* | - |
| *Acholeplasma* | + | Order: ML615J-28 (unclassified genus) | + |
| *TG5* | + | Family: Aerococcaceae (unclassified genus) | - |
| Family: [Mogibacteriaceae] (unclassified genus) | + | *Methanobrevibacter* | + |
| *Mycoplasma* | + | *Pedobacter* | + |
| Order: ML615J-28 (unclassified genus) | + | Class: Bacilli (unclassified genus) | - |
| Family: Leptotrichiaceae (unclassified genus) | + | *BE24* | + |
| Family: Pasteurellaceae (unclassified genus) | - | *Butyrivibrio* | + |
| Family: Pasteurellaceae (unclassified genus) | - | *Peptococcus* | + |
| *Eikenella* | - | Order: Acidimicrobiales (unclassified genus) | - |
| *Desulfobulbus* | + | Family: [Paraprevotellaceae] (unclassified genus) | - |
| *Pseudoramibacter_Eubacterium* | + | Family: Enterobacteriaceae (unclassified genus) | - |
| *Methylobacterium* | - | *Rhizobium* | + |
| *Mogibacterium* | + | *Aerococcus* | - |
| Family: Gemellaceae (unclassified genus) | + | *Filifactor* | + |
| *Scardovia* | - | *Slackia* | - |
| **Shotgun Metagenomics - Taxa** |  |  |  |
| Pre-Treatment |  | Post-Treatment |  |
| *Treponema denticola* | + | *Selenomonas (unclassified species)* | - |
| *Treponema denticola** | + | *Prevotella denticola* | - |
| Phylum: Spirochaetes (unclassified species) | + | *Prevotella denticola** | - |
| Class: Spirochaetia (unclassified species) | + | *Actinomyces Actinomyces oral taxon 448* | - |
| Order: Spirochaetales (unclassified species) | + | *Actinomyces Actinomyces oral taxon 448** | - |
| Family: Spirochaetaceae (unclassified species) | + | *Prevotella oris* | - |
| *Treponema (unclassified species)* | + | *Prevotella oris** | - |
| *Porphyromonas gingivalis* | + | *Mogibacterium (unclassified species)* | + |
| *Porphyromonas gingivalis** | + | *Mogibacterium sp CM50* | + |
| *Treponema socranskii* | + | *Mogibacterium sp CM50** | + |
| Family: Porphyromonadaceae | + | *Eggerthia (unclassified species)* | + |
| *Porphyromonas (unclassified species)* | + | *Eggerthia catenaformis* | + |
| *Treponema socranskii* | + | *Eggerthia catenaformis** | + |
| *Treponema maltophilum* | + | *Dialister (unclassified species)* | - |
| *Treponema maltophilum** | + | *Dialister invisus* | - |
| Phylum: Firmicutes (unclassified species) | - | *Dialister invisus** | - |
| *Tannerella (unclassified species)* | + | Family: Lachnospiraceae (unclassified species) | - |
| *Tannerella forsythia* | + | *Selenomonas sp CM52* | - |
| *Tannerella forsythia** | + | *Selenomonas sp CM52** | - |
| Class: Bacilli (unclassified species) | - | Class: Negativicutes (unclassified species) | - |

**TABLE S4** (cont.)

| **Shotgun Metagenomics - Pathways** |  |  |  |
| --- | --- | --- | --- |
| Pre-Treatment |  | Post-Treatment |  |
| PWY-6853: ethylene biosynthesis II | + | PWY-7297: octopamine biosynthesis | + |
| PROUT-PWY: L-proline degradation | + | PWY-5433: superpathway of lipoxygenase | - |
| GLYSYN-ALA-PWY: glycine biosynthesis III | + | ASPARAGINE-DEG1-PWY: L-asparagine degradation I | - |
| PWY-6616: sulfolactate degradation I | + | PWY-7456: mannan degradation | - |
| PWY-6454: vancomycin resistance I | - | PWY0-1329: succinate to cytochrome bo electron transfer | - |
| PWY-6462: peptidoglycan cross-bridge biosynthesis | - | PWY0-1353: succinate to cytochrome bd electron transfer | - |
| PWY-6463: peptidoglycan cross-bridge biosynthesis | - | BSUBPOLYAMSYN-PWY: spermidine biosynthesis I | - |
| PWY-6841: homophytochelatin biosynthesis | - | METHANOGENESIS-PWY: From H2 and CO2 | + |
| PWY-6117: spermine and spermidine degradation I | - | PWY-2381: 4-nitrobenzoate degradation | + |
| PWY-5724: superpathway of atrazine degradation | + | PWY-1723: formaldehyde oxidation V (H4MPT pathway) | + |
| PWY0-1338: polymyxin resistance | + | PWY-2504: aromatic compound degradation | - |
| PWY-1263: taurine degradation I | + | PWY-6711: archaeosine biosynthesis | + |
| PWY-6366: D-myo-inositol (1,4,5,6)-tetrakisphosphate biosynthesis | - | PWY-5273: p-cumate degradation | - |
| PWY-6365: D-myo-inositol (3,4,5,6)-tetrakisphosphate biosynthesis | - | SORBDEG-PWY: D-sorbitol degradation II | - |
| PWY-5283: L-lysine degradation V | - | METHFORM-PWY: methyl-coenzyme M reduction | + |
| PWY1G-170: formaldehyde oxidation III (mycothiol-dependent) | - | PWY-5823: CDP-glucose-derived O-antigen biosynthesis | - |
| P224-PWY: sulfate reduction V (dissimilatory) | + | POLYAMINSYN3-PWY: polyamine biosynthesis II | - |
| PWY-6436: perillyl aldehyde biosynthesis | + | PWY0-1356: formate electron transfer | + |
| PWY-5418: phenol degradation I (aerobic) | - | PWY-5163: p-cumate degradation to 2-oxopent-4-enoate | - |
| PWY-5755: 4-hydroxybenzoate biosynthesis II | - | PWY-6467: Kdo transfer to lipid IVA III |  |
| **Metabolite Features (MS1)** |  |  |  |
| Pre-Treatment |  | Post-Treatment |  |
| 689.514-689.519_662-666 | - | 1002.083-1002.130_641-708 | + |
| 341.232-341.240_640-653 | - | 549.335-549.344_626-638 | + |
| 609.417-609.428_628-635 | - | 475.095-475.104_625-642 | - |
| 1130.682-1130.697_717-740 | + | 553.386-553.389_734-738 | + |
| 542.387-542.397_642-657 | - | 388.246-388.247_527-530 | + |
| 245.079-245.081_298-305 | - | 739.603-739.609_642-648 | + |
| 682.468-682.496_634-653 | - | 136.054-136.071_235-245 | + |
| 488.328-488.336_699-718 | + | 388.887-388.895_764-804 | + |
| 895.555-895.563_657-679 | - | 192.101-192.104_189-200 | + |
| 465.205-465.213_398-417 | - | 387.237-387.246_29-69 | + |
| 765.414-765.421_440-447 | - | 336.253-336.256_547-552 | + |
| 383.098-383.103_219-232 | - | 1198.674-1198.688_718-740 | + |
| 554.370-554.378_694-707 | + | 931.730-931.755_637-754 | + |
| 295.193-295.195_590-600 | - | 435.175-435.181_415-420 | - |
| 399.211-399.215_642-661 | + | 399.211-399.214_582-586 | + |
| 443.196-443.213_452-469 | - | 531.351-531.371_707-747 | + |
| 716.455-716.461_561-565 | - | 205.129-205.134_766-804 | + |
| 708.463-708.467_620-627 | - | 399.211-399.215_642-661 | + |
| 564.904-564.915_605-638 | - | 341.232-341.240_640-653 | - |
| 479.282-479.299_620-647 | - | 572.394-572.395_668-672 | + |
